# CRB3 regulates contact inhibition by activating the Hippo pathway in mammary epithelial cells

**DOI:** 10.1038/cddis.2016.478

**Published:** 2017-01-12

**Authors:** Xiaona Mao, Pingping Li, Yaochun Wang, Zheyong Liang, Jie Liu, Juan Li, Yina Jiang, Gang Bao, Lei Li, Bofeng Zhu, Yu Ren, Xinhan Zhao, Jianmin Zhang, Yu Liu, Jin Yang, Peijun Liu

**Affiliations:** 1 Center for Translational Medicine, The First Affiliated Hospital of Xi'an Jiaotong University, Xi'an, Shaanxi 710061, P.R. China; 2 Key Laboratory for Tumor Precision Medicine of Shaanxi Province, The First Affiliated Hospital of Xi'an Jiaotong University, Xi'an, Shaanxi 710061, P.R. China; 3 Department of Oncology, The First Affiliated Hospital of Xinxiang Medical University, Weihui, Henan 453100, P.R. China; 4 Department of Neurosurgery, The First Affiliated Hospital of Xi'an Jiaotong University, Xi'an, Shaanxi 710061, P.R. China; 5 Department of Urology, The First Affiliated Hospital of Xi'an Jiaotong University, Xi'an, Shaanxi 710061, P.R. China; 6 Key Laboratory of Shaanxi Province for Craniofacial Precision Medicine Research, College of Stomatology, Xi'an Jiaotong University, Xi'an, Shaanxi 710004, P.R. China; 7 Department of Breast Surgery, The First Affiliated Hospital of Xi'an Jiaotong University, Xi'an, Shaanxi 710061, P.R. China; 8 Department of Oncology, The First Affiliated Hospital of Xi'an Jiaotong University, Xi'an, Shaanxi 710061, P.R. China; 9 Department of Cancer Genetics, Roswell Park Cancer Institute, Elm & Carlton Streets, Buffalo, NY 14263, USA; 10 Department of Biology and Biochemistry, University of Houston, 4039 Leeshire Drive, Houston, TX 77025, USA

## Abstract

The loss of contact inhibition is a hallmark of cancer cells. The Hippo pathway has recently been shown to be an important regulator of contact inhibition, and the cell apical polarity determinant protein CRB3 has been suggested to be involved in Hippo signalling. However, whether CRB3 regulates contact inhibition in mammary cells remains unclear, and the underlying mechanisms have not been elucidated. As shown in the present study, CRB3 decreases cell proliferation, promotes apoptosis, and enhances the formation of tight and adherens junctions. Furthermore, we report for the first time that CRB3 acts as an upstream regulator of the Hippo pathway to regulate contact inhibition by recruiting other Hippo molecules, such as Kibra and/or FRMD6, in mammary epithelial cells. In addition, CRB3 inhibits tumour growth *in vivo*. Collectively, the present study increases our understanding of the Hippo pathway and provides an important theoretical basis for exploring new avenues for breast cancer treatment.

Contact inhibition is a process by which cells stop proliferating when they contact adjacent cells. However, cancer cells do not arrest their growth when they interact with neighbouring cells. Instead, they continue to proliferate, grow on top of each other and form multilayered foci.^1, 2, 3^ The loss of contact inhibition is a hallmark feature of cancer cells.^4^ Therefore, contact inhibition is a powerful anticancer mechanism that prevents cell proliferation. Although a number of signalling pathways have been shown to be involved in contact inhibition, the underlying mechanisms remain poorly understood.

The Hippo pathway plays a crucial role in regulating contact inhibition and is highly conserved in species ranging from Drosophila to mammals as almost all the components of the pathway in Drosophila have recognizable homologues in mammals.^5, 6, 7^ Mst1/2 is activated during the activation of the Hippo pathway in mammals, allowing it to subsequently activate Sav1 and Mob1. Then, Lats1/2 kinases are phosphorylated and activated. Upon the activation of Lats1/2 kinases, the Lats1/2-Mob1 complex in turn phosphorylates YAP/TAZ. Lats1/2 regulate the transcriptional co-activators YAP/TAZ by altering their localization and protein stability. Phosphorylated YAP/TAZ are retained in the cytoplasm when bound to 14-3-3 and are degraded by *β*-TrCP.^8, 9, 10^ Upon inactivation of the Hippo pathway, the dephosphorylated YAP/TAZ translocate to the nucleus, where they bind to transcription factors such as TEAD and SMAD.^11, 12^ In addition to these downstream regulators, multiple upstream regulators of the Hippo pathway have been identified in Drosophila, including WW and C2 domain containing 1 (WWC1/Kibra, homologue of mammalian Kibra),^13, 14, 15^ Merlin (homologue of mammalian NF2) and Expanded (homologue of mammalian FRMD6).^16, 17, 18^ However, the upstream regulators have not been studied as intensively as the kinase cascade in mammals.

Apical-basal polarity components, particularly the apical polarity determinant Crb, have recently been suggested to regulate Hippo pathway activity in Drosophila.^19^ Three apical-basal polarity complexes have been identified to date, including the CRB (Crumbs) complex (CRB/PATJ/PALS1), PAR complex (PAR3/PAR6/aPKC) and Scribble complex (SCRIB/DLG/LGL). These complexes work together to maintain apical-basal polarity.^20^ Mammals have three Crumbs family members (CRB1-3).^21^ In Drosophila, Crb directly interacts with the upstream Hippo pathway component Expanded.^22, 23, 24^ In mammals, the depletion of CRB3 leads to increased nuclear localization of YAP/TAZ,^25, 26^ suggesting that CRB3 is associated with the Hippo pathway. Little is currently known about the functional roles of CRB3 in mammals. Furthermore, CRB3 is associated with contact inhibition.^21^ Nevertheless, the detailed molecular mechanisms linking CRB3 to the regulation of the Hippo pathway and contact inhibition are still not clear.

In this study, we aimed to investigate the precise mechanisms by which CRB3 regulates the Hippo pathway and contact inhibition. Based on our observations, CRB3 acted as an upstream regulator of the Hippo pathway and regulated contact inhibition via Kibra and/or FRMD6.

## Results

### CRB3 is an important mediator of contact inhibition

We examined CRB3 expression in breast cancer tissues and cells to investigate the role of CRB3 in breast cancer. CRB3 expression was low in breast cancer tissues but was high in adjacent normal breast tissues (Supplementary Figures S1a–c). CRB3 expression was correlated with tumour size but not with age, histological grade, clinical stage or lymph node involvement (Supplementary Table S1). The expression levels of the CRB3 mRNA and protein were significantly lower in breast cancer cells than in immortalized mammary epithelial cells (Supplementary Figures S1d and e). Our results may provide a clue about the relationship between the loss of CRB3 expression and the loss of contact inhibition, a hallmark of cancer cells.^4^

**Figure 1:**
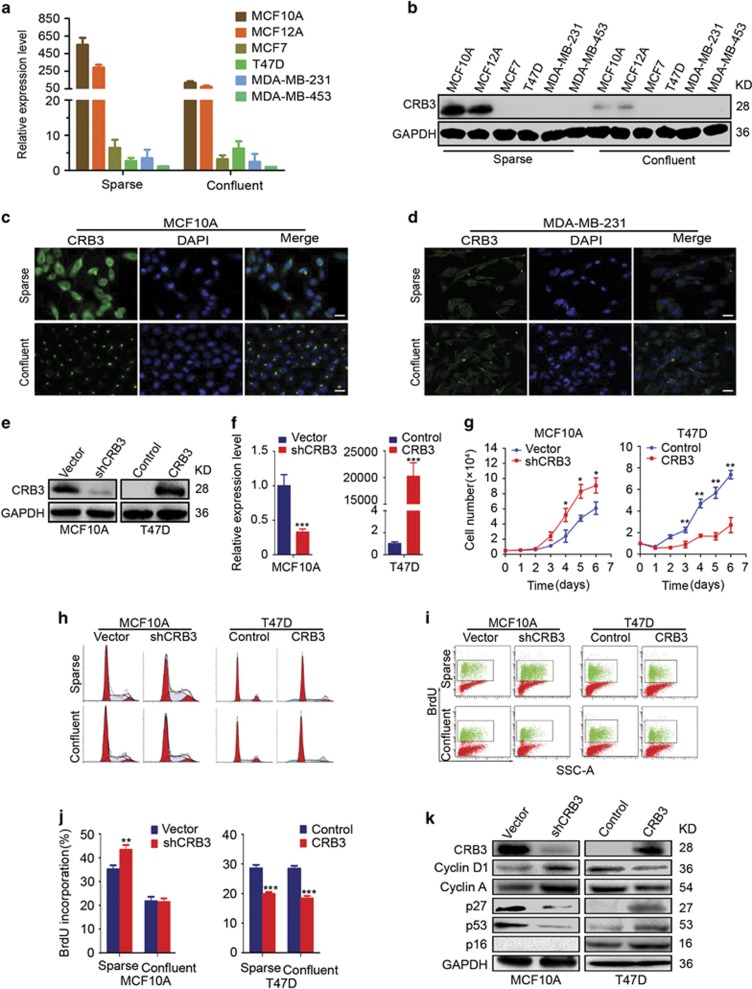
CRB3 is an important mediator of contact inhibition. (**a, b**) Expression of CRB3 mRNA and protein was examined in cells grown under sparse and confluent conditions by real-time PCR and immunoblot analyses, respectively. GAPDH was used as a loading control. (**c, d**) The localization of CRB3 in MCF10A and MDA-MB-231 cells grown under sparse and confluent conditions was determined using immunofluorescence. Scale bar, 25 *μ*m. (**e** and **f**) The efficiencies of CRB3 knockdown and upregulation were determined by immunoblot analysis and real-time PCR. ***P<0.001. Data are mean±S.D. (**g**) The proliferation of MCF10A and T47D cells was evaluated for six successive days using a cell proliferation assay. *P<0.05, **P<0.01. Data are mean±S.D. (**h**) A cell cycle analysis was used to measure the cell cycle distribution of cells grown under sparse and confluent conditions. (**i, j**) BrdU incorporation was examined to reveal DNA synthesis in cells grown under sparse and confluent conditions. **P<0.01, ***P<0.001. Data are mean±S.D. (**k**) The protein expression levels in the indicated cells were detected by immunoblotting

CRB3 expression was examined in cells grown under sparse and confluent conditions to determine the relationship between CRB3 expression and contact inhibition. CRB3 expression was significantly lower in breast cancer cells than in immortalized mammary epithelial cells, regardless of cell density (Figures 1a and b). Interestingly, CRB3 expression was significantly lower in confluent MCF10A and MCF12A cells than in the sparse cells, but further studies are needed to investigate the mechanisms underlying the contact-dependent change in CRB3 expression. The localization of CRB3 is essential for the maintenance of cell polarity. Immunofluorescence staining for CRB3 showed that the protein was predominantly expressed in the membrane and cytoplasm of sparse MCF10A cells and sparse and confluent MDA-MB-231 cells, but CRB3 was predominantly localized in a certain region of the membrane of confluent MCF10A cells (Figures 1c and d). The different localization patterns of CRB3 in confluent cell cultures may suggest that its function varies among different cells.

CRB3 was knocked down in MCF10A cells and overexpressed in T47D and MDA-MB-453 cells (Figures 1e and f and Supplementary Figures S2a–b). Cell proliferation assays, cell cycle analyses and BrdU incorporation assays were performed to examine the effects of CRB3 on cell proliferation. CRB3 knockdown accelerated G1/S progression and increased BrdU incorporation and cell proliferation in MCF10A cells grown under sparse conditions, whereas CRB3 overexpression decreased BrdU incorporation and cell proliferation in T47D and MDA-MB-453 cells, regardless of cell density (Figures 1g–j, Supplementary Figures S3a–b and Supplementary Figures S2c–e). However, CRB3 overexpression in T47D cells had no effect on the cell cycle, except that it induced the appearance of a pre-G1 peak (Figure 1h and Supplementary Figures S3c). Thus, CRB3 plays an important role in contact inhibition. Moreover, the expression levels of p27 and p53, two regulators of contact inhibition,^27^ were decreased in shCRB3 cells (Figure 1k). In addition, p16, another regulator of contact inhibition,^27, 28^ was not expressed because MCF10A cells may have p16 deletion, as previously reported by Debnath J *et al.*^29^ The levels of p27, p53 and p16 were increased when CRB3 was overexpressed in T47D and MDA-MB-453 cells (Figure 1k and Supplementary Figure S2f), suggesting that p16, p27 and p53 play crucial roles in CRB3-mediated regulation of contact inhibition. Cyclin D1 and Cyclin A expression were increased in CRB3 knockdown cells but reduced in CRB3-overexpressing T47D and MDA-MB-453 cells (Figure 1k and Supplementary Figure S2f). Thus, CRB3 is an important factor that regulates contact inhibition.

### CRB3 overexpression promotes breast cancer cell apoptosis

**Figure 2:**
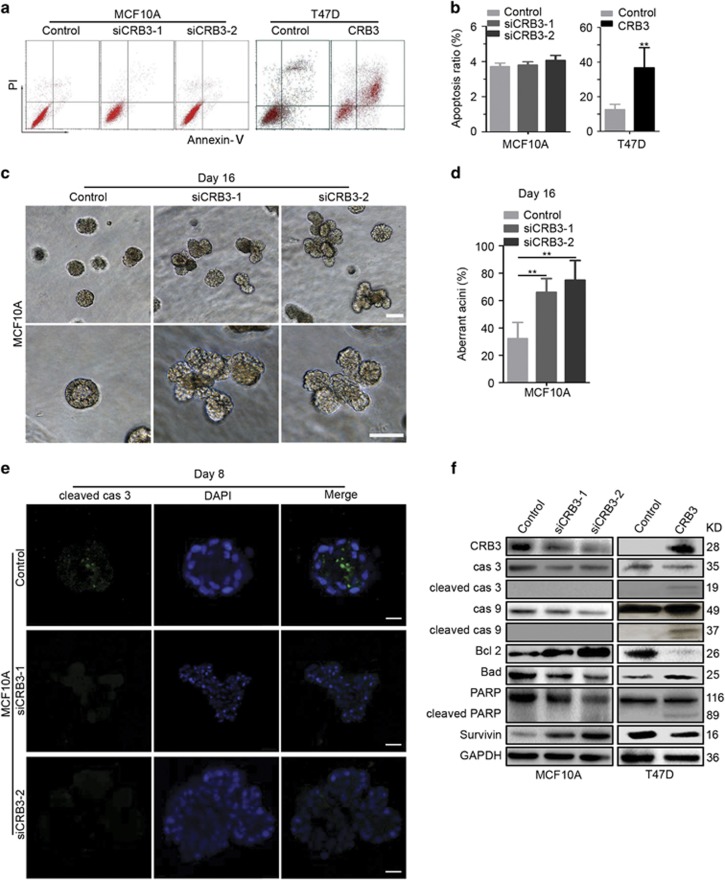
CRB3 overexpression promotes breast cancer cell apoptosis. (**a, b**) Apoptosis of MCF10A and T47D cells was examined using an apoptosis assay. **P<0.01. Data are mean±S.D. (**c, d**) 3D cultures were used to reveal the formation of acini of MCF10A cells. The images were photographed at the indicated times. Scale bar, 50 *μ*m.**P<0.01. Data are mean±S.D. (**e**) Cleaved caspase-3 (cleaved cas 3) staining was performed on 3D cultures of MCF10A cells to examine the cells undergoing apoptosis. Scale bar, 25 μm. (**f**) An immunoblot analysis was used to detect the expression of apoptosis-related proteins. Cas 3, caspase 3; cas 9, caspase 9

We further investigated whether CRB3 overexpression affected cell apoptosis to determine whether contact inhibition was mediated by increased apoptosis or decreased cell proliferation. CRB3 knockdown with siRNA in MCF10A cells had no effect on cell apoptosis (Figures 2a and b). However, CRB3 overexpression in T47D and MDA-MB-453 cells caused a significant increase in apoptosis (Figures 2a and b and Supplementary Figures S4a–b), suggesting that CRB3 overexpression induced cell apoptosis. A three-dimensional (3D) culture model is more suitable than cell monolayers to describe cell apoptosis in MCF10A cells because those can form polarized acinar structures in 3D cultures that are similar to the formation of mammary acini *in vivo*.^29^ Therefore, we established a 3D cell culture system to detect apoptosis. Negative control cells formed acini with hollow lumens, whereas CRB3 knockdown (siCRB3-1 and siCRB3-2) resulted in the formation of much larger acini and multiacinar structures (Figures 2c and d). An analysis of cells undergoing apoptosis showed cleaved caspase-3 staining in negative control cells but no staining in CRB3 knockdown cells in 3D cultures (Figure 2e), indicating that CRB3 plays a critical role in regulating the formation of acini. Caspase 3/9, PARP and other apoptosis-related proteins were examined to reveal the detailed mechanism by which CRB3 induced apoptosis. We observed increased levels of the cleaved caspase 3/9 and PARP proteins in CRB3-overexpressing T47D and MDA-MB-453 cells but the levels of these proteins were not increased in the MCF10A cells (Figure 2f and Supplementary Figure S4c). The expression levels of Bcl2 and Survivin were increased and Bad was decreased when CRB3 expression was inhibited in MCF10A cells (Figure 2f). By contrast, Bcl2 and Survivin expression were decreased and Bad expression was increased in CRB3-overexpressing T47D and MDA-MB-453 cells (Figure 2f and Supplementary Figure S4c). Thus, CRB3 may regulate contact inhibition by promoting cell apoptosis.

### The loss of CRB3 disrupts the formation of tight junctions and adherens junctions

**Figure 3:**
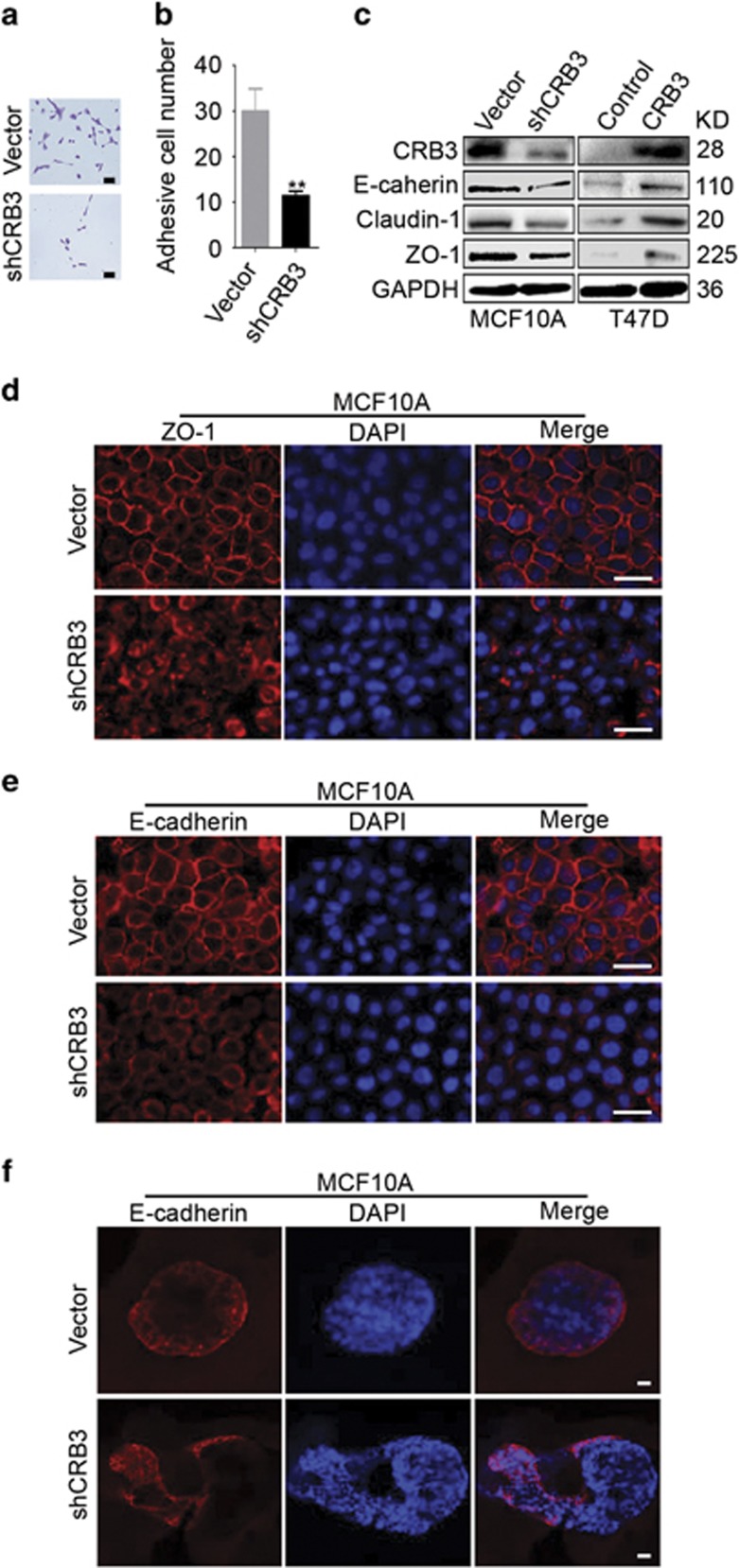
The loss of CRB3 disrupts the formation of tight and adherens junctions. (**a, b**) A cell adhesion assay was performed to detect the adhesion of MCF10A cells. Scale bar, 50 *μ*m **P<0.01. Data are mean±S.D. (**c**) The levels of the ZO-1, Claudin-1 and E-cadherin proteins were determined by immunoblotting. (**d, e**) The localization of ZO-1 and E-cadherin was examined in MCF10A cells grown in 2D culture. Scale bar, 25 *μ*m. (**f**) The localization of E-cadherin in 3D cultures was detected using immunofluorescence staining. Scale bar, 25 *μ*m

Cell–cell contacts, including tight and adherens junctions,^21, 30^ play a crucial role in the maintenance of apical-basal polarity and contact inhibition.^5, 31, 32^ CRB3 knockdown significantly reduced the adhesion of MCF10A cells in a cell adhesion assay (Figures 3a and b). An immunoblot analysis was performed to determine the expression levels of the tight junction proteins Claudin-1 and ZO-1 and the adherens junction protein E-cadherin. The expression levels of the three proteins were reduced upon CRB3 knockdown, but their expression increased upon CRB3 overexpression (Figure 3c). We investigated the effects of CRB3 knockdown on the localization of ZO-1 and E-cadherin to verify this result. Staining for ZO-1 and E-cadherin in vector control cells revealed a smooth, continuous and well-defined staining pattern at the membranes between cells (Figures 3d and e). By contrast, CRB3 knockdown cells displayed diffuse ZO-1 and E-cadherin staining patterns, suggesting impaired formation of tight and adherens junctions (Figures 3d and e). The 3D culture assay yielded consistent results for E-cadherin localization (Figure 3f). Based on these results, CRB3 regulated contact inhibition by affecting the formation of tight and adherens junctions.

### CRB3 is an upstream regulator of the Hippo pathway and affects FRMD6 transcription

**Figure 4:**
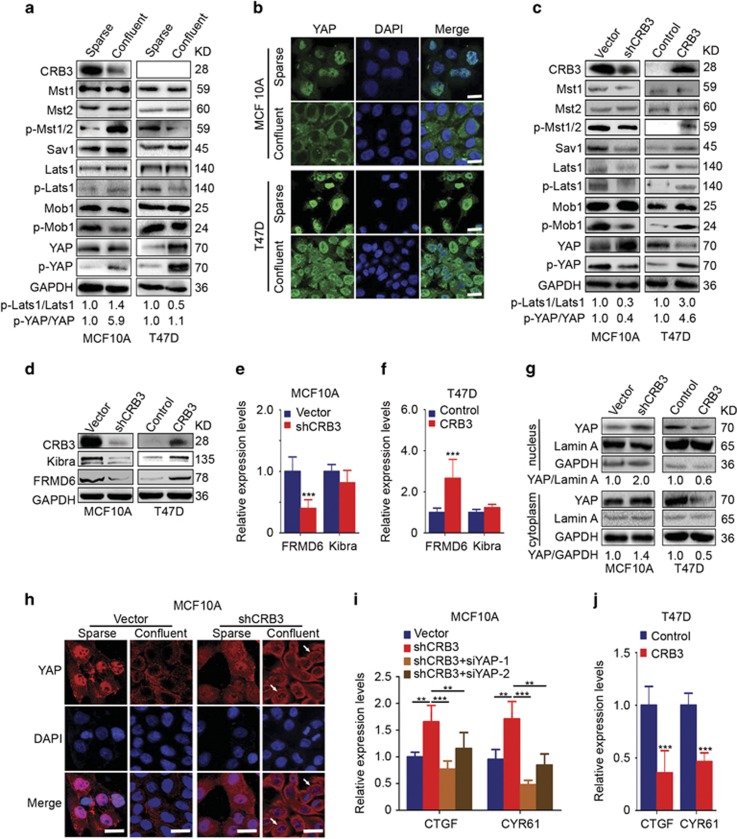
CRB3 is an upstream regulator of the Hippo pathway and affects FRMD6 transcription. (**a**) Protein levels of Hippo pathway components were examined in cells grown under sparse and confluent conditions. The numbers below the images indicate the relative expression levels of p-Lats1/Lats1 and p-YAP/YAP. (**b**) YAP localization was assessed in cells grown under sparse and confluent conditions. Scale bar, 25 *μ*m. (**c**) Protein expression of the core regulators of the Hippo pathway was examined by immunoblotting. The numbers below the images represent relative expression levels of p-Lats1/Lats1 and p-YAP/YAP. (**d-f**) The expression levels of the FRMD6 and Kibra proteins and mRNAs were analysed by immunoblotting (**d**) and real-time PCR (**e** and **f**). ***P<0.001. Data are mean±S.D. (**g**) Cellular fractionation experiments were performed to examine the levels of the CRB3 protein in the nucleus and cytoplasm. GAPDH and Lamin A were used as loading controls. The numbers below the images indicate the relative expression level of YAP. (**h**) YAP localization was assessed in MCF10A cells grown under sparse and confluent conditions. Scale bar, 25 *μ*m. (**i, j**) The expression levels of the CTGF and CYR61 mRNAs in MCF10A and T47D cells were examined using real-time PCR. shCRB3 cells were transfected with siRNAs to silence the expression of YAP. Cells were collected 48 h post-transfection. **P<0.01, ***P<0.001. Data are mean±S.D.

The Hippo pathway is implicated in the regulation of contact inhibition.^5, 6, 7^ CRB3 has previously been shown to be associated with contact inhibition and regulates the Hippo pathway effector YAP.^21, 25, 26^ Based on these reports, we wondered whether CRB3 was an upstream regulator of the Hippo pathway and whether CRB3 regulated contact inhibition via the Hippo pathway. We first examined the protein expression levels of components of the Hippo pathway in MCF10A and T47D cells grown under sparse and confluent conditions. CRB3 expression was reduced and p-Mst1/2, p-Lats1 and p-YAP expression were increased in cells grown under confluent conditions compared with expression in MCF10A cells grown under sparse conditions (Figure 4a). CRB3 expression was not detected and the YAP and p-YAP expression levels were apparently increased in T47D cells grown under confluent conditions (Figure 4a). The expression levels of Mst1, Mst2, Sav1, Lats1 and Mob1 were not significantly altered in MCF10A and T47D cells (Figure 4a). Lats2 expression was not detected in MCF10A cells since it does not functionally antagonize YAP in these cells.^33^ As shown in the immunofluorescence analysis of MCF10A cells, YAP was localized to the nucleus in sparse cells and to the cytoplasm in confluent cells (Figure 4b). The YAP protein predominantly remained in the nucleus of T47D cells, even under confluent conditions (Figure 4b). Thus, the Hippo pathway was active in confluent MCF10A cells but was inactive in confluent T47D cells. We then studied the effects of CRB3 on the Hippo pathway. The levels of phosphorylated Mst1/2, Lats1, Mob1 and YAP were decreased, but no changes in the expression of Mst1, Mst2, Lats1 and Mob1 were detected in shCRB3 cells (Figure 4c). By contrast, the levels of phosphorylated Mst1/2, Lats1, Mob1 and YAP were increased in the CRB3-overexpressing cells (Figure 4c). Moreover, p-YAP expression was reduced when Mst2 or Lats1 were knocked down with siRNA in shCRB3 cells (Supplementary Figures S5a and b). The levels of p-Lats1 were decreased when Mst2 was silenced in shCRB3 cells (Supplementary Figure S5a). Thus, CRB3 promoted YAP phosphorylation through the canonical Hippo pathway. The proteins assessed here were proteins downstream of the Hippo pathway, suggesting that CRB3 may regulate the Hippo pathway via upstream proteins.

Kibra and FRMD6, upstream regulators of the Hippo pathway, were previously suggested to regulate the Hippo pathway in MCF10A cells, but NF2 does not act in the same manner.^34, 35^ Therefore, only FRMD6 and Kibra expression were examined in this study. The levels of the FRMD6 mRNA and protein were downregulated in shCRB3 cells and up-regulated in CRB3-overexpressing cells (Figures 4d–f). The expression of the Kibra protein was positively correlated with CRB3 expression; however, CRB3 had no effect on the expression of the Kibra mRNA (Figures 4d–f). Thus, CRB3 is an upstream regulator of the Hippo pathway and affects FRMD6 transcription.

As shown in the cellular fractionation experiments, nuclear YAP expression was increased in the shCRB3 cells whereas YAP expression was decreased in the CRB3-overexpressing T47D cells (Figure 4g). YAP was localized in the cytoplasm in confluent vector control cells, whereas YAP was localized in the nucleus and cytoplasm in shCRB3 cells, regardless of cell density (Figure 4h). The expression levels of the CTGF and CYR61 mRNAs, two YAP target genes,^36, 37^ were increased in the shCRB3 cells and inhibited by YAP silencing in the shCRB3 cells (Figure 4i), suggesting that the function of CRB3 may depend on YAP expression. Effective knockdown of YAP expression was confirmed by real-time PCR (Supplementary Figure S6). However, the expression of the CTGF and CYR61 mRNAs was decreased in the CRB3-overexpressing cells (Figure 4j). Thus, CRB3 regulates YAP localization in response to cell density and CRB3 is an upstream regulator of the Hippo pathway.

### CRB3 stabilizes Kibra by inhibiting its degradation, and Kibra rescues cell proliferation induced by CRB3 deregulation

**Figure 5:**
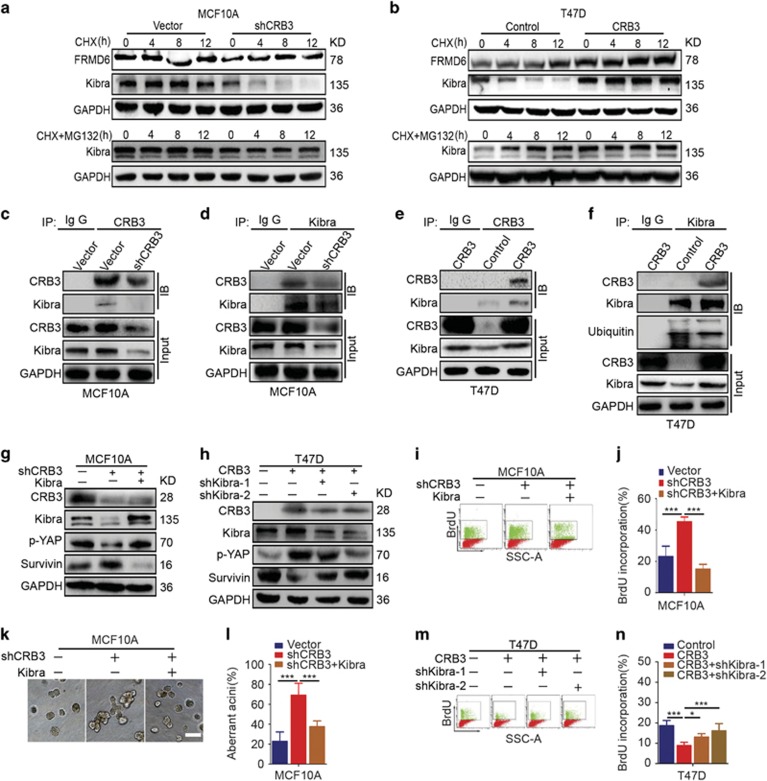
CRB3 stabilizes the Kibra protein by inhibiting its degradation, and Kibra rescues the changes in cell proliferation induced by CRB3 deregulation. (**a**) MCF10A cells were treated with cycloheximide (100 *μ*g/ml) and MG132 (5 *μ*M) for the indicated times. Cell lysates were examined for the levels of the FRMD6 and Kibra proteins by immunoblotting. (**b**) T47D cells were treated with cycloheximide (100 *μ*g/ml) and MG132 (5 *μ*M) for the indicated times. The levels of the FRMD6 and Kibra proteins were determined by immunoblotting. (**c, d**) MCF10A cell lysates were subjected to immunoprecipitation experiments with an anti-CRB3 or anti-Kibra antibody. Immunoblot analyses were conducted for the indicated proteins. (**e, f**) Immunoprecipitation experiments were performed in T47D cells. (**g, h**) The protein levels were detected by immunoblotting. (**i**, j) DNA synthesis was examined in MCF10A cells using BrdU incorporation. ***P<0.001. Data are mean±S.D. (**k** and **l**) Aberrant formation of acini was detected in 3D cultures of MCF10A cells. Scale bar, 50 *μ*m.***P<0.001. Data are mean±S.D. (**m, n**) BrdU incorporation was assessed to examine DNA synthesis in T47D cells. *P<0.05, ***P<0.001. Data are mean±S.D.

A significant change in Kibra mRNA expression was not observed in CRB3-overexpressing cells; thus, we hypothesized that the increased expression of the Kibra protein may be due to the stabilization of the protein. We first measured the half-lives of Kibra and FRMD6 in the context of loss or overexpression of CRB3 to verify this hypothesis. The stability of the FRMD6 protein was not altered by the presence or absence of CRB3 (Figures 5a and b). CRB3 knockdown decreased the half-life of Kibra, whereas CRB3 overexpression significantly prolonged the half-life of Kibra, indicating that CRB3 stabilized the Kibra protein (Figures 5a and b). Like many other proteins, Kibra is degraded through the proteasome pathway. Because the proteasome inhibitor MG132 significantly inhibited the decrease in the levels of the Kibra protein in CRB3 knockdown MCF10A cells and T47D control cells (Figures 5a and b). As shown in immunoprecipitation experiments, CRB3 interacted with Kibra (Figures 5c–f). Interestingly, Kibra was polyubiquitinated in control T47D cells and not in CRB3-overexpressing cells (Figure 5f), suggesting that CRB3 stabilized the Kibra protein by inhibiting its degradation through the ubiquitin-proteasome pathway.

Kibra has previously been shown to regulate YAP phosphorylation.^38^ We hypothesized that the aforementioned phenotype was caused by Kibra-induced YAP phosphorylation. We measured the levels of p-YAP and its downstream target genes by immunoblotting to confirm this hypothesis. We used a plasmid to overexpress Kibra in the CRB3 knockdown cells because Kibra expression was decreased in MCF10A cells upon CRB3 silencing. Interestingly, Kibra overexpression reversed the expression patterns of p-YAP and the YAP target gene Survivin^39^ (Figure 5g). Two shRNAs were used to silence Kibra expression because Kibra expression was increased in CRB3-overexpressing T47D cells (Figure 5h). Upregulation of CRB3 increased YAP phosphorylation and reduced Survivin expression, and these alterations were partially reversed upon Kibra silencing (Figure 5h). Based on these results, YAP phosphorylation was mediated by Kibra, and CRB3 exhibited crosstalk with Kibra in the Hippo pathway. Furthermore, Kibra overexpression significantly inhibited BrdU incorporation (Figures 5i and j) and partially rescued the aberrant formation of MCF10A acini (Figures 5k and l) induced by CRB3 knockdown. By contrast, increased BrdU incorporation was observed when Kibra was silenced in CRB3-overexpressing T47D cells (Figures 5m and n). Overall, Kibra rescued cell proliferation and the aberrant formation of acini induced by CRB3 deregulation.

### CRB3 inhibits tumour growth *in vivo*

**Figure 6:**
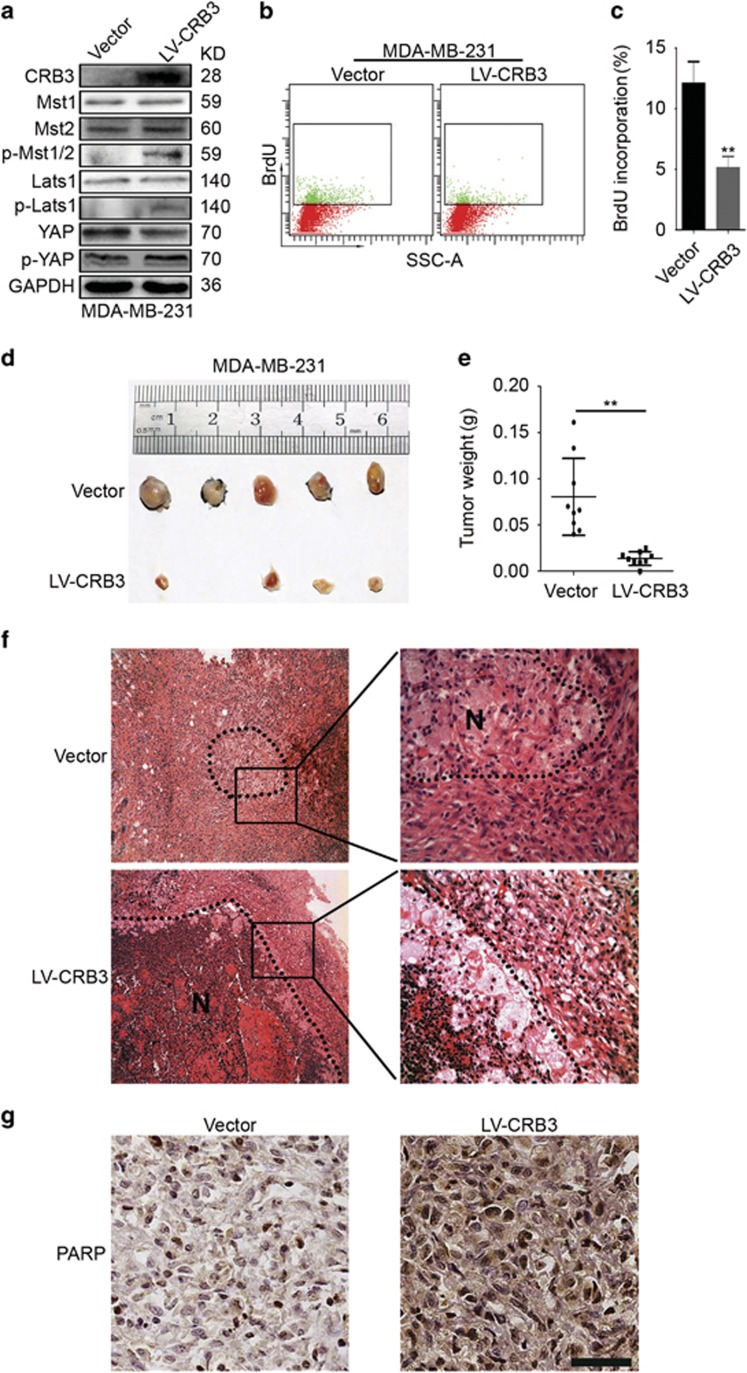
CRB3 inhibits tumour growth *in vivo*. (**a**) MDA-MB-231 cells were infected with a lentivirus (LV-CRB3) overexpressing CRB3. Immunoblots were performed to examine the efficiency of CRB3 overexpression and the expression levels of Mst1, Mst2, p-Mst1/2, Lats1, p-Lats1, YAP and p-YAP. (**b, c**) A BrdU incorporation analysis was performed to assess the effects of CRB3 on the proliferation of MDA-MB-231 cells. **P<0.01. Data are mean±S.D. (**d**) SCID mice bearing human breast cancer xenografts were maintained for 25 days. Representative images of the tumours are shown. (**e**) Tumours were dissected and the tumour weights were plotted. **P<0.01. Data are mean±S.D. (**f**) Hematoxylin-eosin staining of the tumour specimens is shown. N, necrosis. (**g**) PARP expression in the tumour tissues was examined using immunohistochemistry. Scale bar, 37 *μ*m

CRB3 overexpression inhibited breast cancer cell proliferation and induced cell apoptosis *in vitro*. We wondered whether CRB3 inhibited tumour growth *in vivo*. The levels of p-Mst1/2, p-Lats1 and p-YAP were increased and the levels of YAP were decreased in CRB3-overexpressing MDA-MB-231 cells (Figure 6a). Meanwhile, BrdU incorporation was decreased (Figures 6b and c). Based on these results, CRB3 reduced the proliferation of MDA-MB-231 cells. In the xenograft studies, the tumour volumes and weights were reduced in the LV-CRB3 group (Figures 6d and e). Necrosis of the LV-CRB3 tumours was increased compared with that of the vector control tumours (Figure 6f). We speculated that necrosis may be due to the increased cell apoptosis because the expression of the apoptosis-related protein PARP was increased in the LV-CRB3 tumours around the necrotic tissue (Figure 6g). Based on these data, CRB3 decreased tumour growth and increased tumour cell apoptosis *in vivo*.

## Discussion

The loss of contact inhibition is a hallmark of cancer cells.^4^ The Hippo pathway is an important regulator of cell-contact inhibition.^5, 6, 7^ A very close relationship between polarity proteins and Hippo pathway components has been established over the past few years.^5, 40^ CRB3 has been shown to regulate growth arrest via contact inhibition.^21^ Additionally, Crb acts as an upstream regulator of the Hippo pathway in Drosophila.^5^ However, researchers have not yet determined whether CRB3 (the homologue of Drosophila Crb) regulates contact inhibition through the Hippo pathway. In the present study, CRB3 regulated contact inhibition via the upstream regulators of the Hippo pathway Kibra and/or FRMD6.

Interestingly, CRB3 expression was significantly higher in immortalized mammary epithelial cells grown under sparse conditions than in cells grown under confluent conditions. We speculate that this difference in CRB3 expression might be due to CRB3 endocytosis or recycling. In Drosophila, cis or trans interactions have been observed between Crbs, and Crb may form homophilic bonds at the epithelial cell surface.^41^ Crb expression is also regulated by Retromer or endocytosis.^42, 43^ CRB3 is the homologue of Crb in mammals, indicating that CRB3 may also be regulated by endocytosis or Retromer in mammalian cells (Supplementary Figure S7). Meanwhile, CRB3 may be located at the epithelial cell surface by participating in a homophilic bond. CRB3 may act as an upstream regulator that is capable of transducing the Hippo pathway signals (Supplementary Figure S7). However, few studies have been performed on the mechanism of CRB3 metabolism in mammals. Furthermore, the proliferation of MCF10A cells grown under confluent and sparse conditions was not different in our study. One possible explanation is that CRB3 was expressed at significantly higher levels in MCF10A cells than in other breast cancer cells. Although most CRB3 was silenced, the remaining CRB3 may have been sufficient to maintain its biological function.

**Figure 7:**
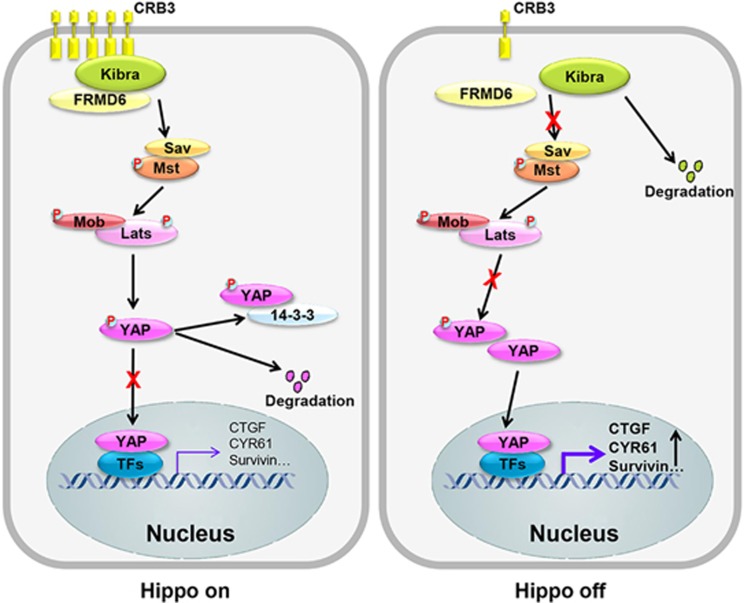
The model shows CRB3-mediated regulation of the Hippo pathway. CRB3 interacts with Kibra to stabilize Kibra expression, which induces YAP phosphorylation and leads to YAP retaining in the cytoplasm. The Hippo pathway is activated (Hippo on), the expressions of YAP target genes are inhibited, cell proliferation is decreased and cell apoptosis is increased. When CRB3 expression is low, the Hippo pathway is inactivated (Hippo off), inducing the expressions of YAP target genes, increasing cell proliferation and decreasing cell apoptosis. TFs, transcription factors

The Hippo pathway effector YAP has recently been shown to regulate contact inhibition by controlling cell proliferation and cell survival.^36, 44^ Several regulatory mechanisms underlying this phenomenon have been reported, including transcriptional upregulation of Survivin or downregulation of cell cycle progression factors, such as Cyclin D1 and Cyclin A.^36, 39^ As shown in this study, the loss of CRB3 increased the level of YAP in the nucleus and increased the levels of YAP target genes^36, 37, 45^ such as Survivin, CTGF and CYR61. We also used the Gene Expression Omnibus database to analyse the correlations between CRB3 and YAP, Kibra, and other proteins of the Hippo pathway. One recent study analysed the relationships between CRB3 expression and the expression of YAP, Kibra, and other proteins in the pathway.^46^ The researchers of the study found that apical localization of CRB3 in differentiated daughter cells correlates with the cytoplasmic localization of YAP, and Kibra co-localized with CRB3. Our results were consistent with the observations. Furthermore, CRB3 regulates contact inhibition through the Hippo pathway regulators Kibra or/and FRMD6 and acts as an upstream regulator of the Hippo pathway. Upon CRB3 overexpression, Kibra was not degraded through the ubiquitin-proteasome system, the Hippo pathway was activated, cell proliferation was decreased and cell apoptosis was increased (Figure 7). The opposite results were obtained when CRB3 was silenced (Figure 7). However, the precise mechanism by which CRB3 stabilizes Kibra is currently unclear. Identification of the specific E3 ligase(s) and F-Box proteins involved in Kibra ubiquitination and proteasomal degradation will be highly informative. In addition, YAP may play an opposite role in breast cancer. As shown in most reports, YAP acts as an oncogene;^47, 48, 49^ however, other reports suggest that YAP may also function as a tumour suppressor.^50, 51, 52^ Thus, the roles of YAP are complicated, and further studies are required to determine the precise mechanisms underlying YAP function in breast cancer.

Polarity proteins and junction proteins regulate each other^30^ and tight junction and adherens junction proteins regulate the Hippo pathway in mammalian cells.^19, 32, 53^ In this study, the loss of CRB3 also resulted in the deregulation of tight junctions and adherens junctions, implying that crosstalk likely exists between different regulatory branches. Interestingly, Kibra has been shown to interact with PATJ, a member of the CRB complex.^54^ Therefore, we cannot exclude the possibility that a CRB3-PALS1-PATJ-Kibra complex exists, and the function of CRB3 may be regulated through Kibra or its associated proteins.

YAP overexpression leads to the loss of contact inhibition and YAP deregulation has been identified in various types of cancer.^8^ As shown in recent studies, YAP may be a therapeutic target for cancer.^8, 55^ CRB3 is a tumour suppressor and is expressed at low levels in cancer tissues.^21, 56^ Furthermore, CRB3 inhibited the function of YAP *in vitro*. In addition, CRB3 decreased cell proliferation and increased cell apoptosis *in vitro* and *in vivo*. Based on these data, CRB3 overexpression may be a therapeutic approach for breast cancer treatment.

## Materials and methods

### Cell culture, transfection and lentiviral infection

All cell lines were purchased from Shanghai Institute of Biochemistry and Cell Biology, Chinese Academy of Sciences (Shanghai, China). MCF7, T47D, MDA-MB-231 and MDA-MB-453 cells were maintained in DMEM medium (Hyclone, Logan, UT, USA) supplemented with 10% FBS (Hyclone) and 1% Penicillin–Streptomycin (Hyclone). MCF10A cells were cultured as previously described.^57^ MCF12A cells were cultured in the same way as the MCF10A cells. The cells were incubated in 5% CO_2_ at 37 °C.

T47D cells were transfected with GV168-CRB3 plasmid (Shanghai Genechem Biotechnology, China) to overexpress CRB3 using TurboFect Transfection Reagent (Thermo Fisher Scientific, Carlsbad, CA, USA) according to the manufacturer's instructions. Twelve hours after transfection, CRB3 overexpressing cells were transfected with pLKO.1 lentiviral shRNA plasmids (shKibra) to knock down Kibra using TurboFect Transfection Reagent.

MCF10A cells were transfected with siRNA to silence CRB3 expression using Lipofectamine 2000 (Thermo Fisher Scientific) according to the manufacturer's instructions. The target sequences of CRB3 siRNA oligonucleotides purchased from Shanghai GenePharma were as follows: siCRB3-1, 5′-AUGAGAAUAGCACUGUUUUTT-3′ siCRB3-2, 5′-UGGCACUGUUGGUGCGGAATT-3′ Negative control (Non-targeting), 5′-UUCUCCGAACGUGUCACGUTT-3′. CRB3-downregulated stable cell lines were generated by infecting MCF10A cells with lentiviral shRNA (shCRB3, Shanghai GenePharma, China) and vector control lentivirus in the presence of 5 μg/ml polybrene (Shanghai GenePharma). Cells were selected for 1 week using 2 μg/ml puromycin (Sigma-Aldrich, Louis, MO, USA). The shCRB3 sequence was as follows: GGGCAAATACAGACCACTTCT. shCRB3 cells were transfected with lentiviral plasmid (Kibra) using TurboFect Transfection Reagent to overexpress Kibra. shCRB3 cells were transfected with siRNA (Shanghai GenePharm) to silence YAP, Mst2 or Lats1 using Lipofectamine 2000 (Thermo Fisher Scientific) according to the manufacturer's instructions. The target sequences of YAP siRNA oligonucleotides were as follows: siYAP-1, 5′-GGUGAUACUAUCAACCAAATT-3′ siYAP-2, 5′-GACGACCAAUAGCUCAGAUTT-3′ siMst2-1, 5′-GCCCAUAUGUUGUAAAGUATT -3′ siMst2-2, 5′-GCUGGUCAGUUAACAGAUATT-3′ siMst2-3, 5′-CCCACAAAUCCACCACCAATT-3′ siLats1-1, 5′- GCCGGCAAAUGUUACAAGATT-3′ siLats1-2, 5′-GAGCUGGAAAGGUUCUAAATT-3′ siLats1-3, 5′-GCAGCGUCUACAUCGUAAATT-3′.

MDA-MB-231 cells were infected with lentivirus (LV-CRB3, Shanghai GenePharma) or vector control lentivirus to overexpress CRB3 using the same method as MCF10A cells were infected, except that 1 μg/ml puromycin was adopted.

The knockdown efficiency of CRB3 in MCF10A cells and the overexpression efficiency of CRB3 in MDA-MB-231 cells were quantified by real time-PCR and/or immunoblot analysis. Efficiency of transient transfection was examined by immunoblot analysis 48 h after transfection.

### Immunoblot analysis, immunoprecipitation experiments and cell fractionation assays

Antibodies Mst1 (#3682), Mst2 (#3952), p-Mst1/2 (#3681), Sav1 (#13301), Lats1 (#3477), phosphor-Lats1 (Thr1079, #8654), Mob1 (#3863), phosphor-Mob1 (Thr35, #8699), YAP (#4912), phospho-YAP (Ser127, #13008), PARP (#9532), caspase-3 (#9665), cleaved caspase-3 (#9664), caspase-9 (#9508), cleaved caspase-9 (#7237), ubiquitin (#3936), claudin-1 (#13255), Kibra (#8774) and FRMD6 (#14688) were obtained from Cell Signaling Technology (Beverly, MA, USA). Antibodies CRB3 (sc-292449), p27 (sc-528), cyclin A (sc-751) and Bcl2 (sc-492) were purchased from Santa Cruz Biotechnology (Santa Cruz, CA, USA). Antibodies GAPDH (HRP-6004), cyclin D1 (60186-1-Ig), p16 (10883-1-AP), Survivin (10508-1-AP), p53 (10442-1-AP), Bad (10435-1-AP) and Lamin A (10298-1-AP) were obtained from Proteintech Group Inc. (Wuhan, China). E-cadherin (ab1416) antibody was purchased from Abcam (Cambridge, MA, USA). ZO-1(339100) antibody was obtained from Thermo Fisher Scientific. Total cell lysate preparation and immunoblot analysis were done as previously described.^56^ For immunoprecipitation experiments, proteins were extracted from cells using immunoprecipitation lysis buffer (20 mM Tris-HCl (pH 8.0), 20% glycerol, 150 mM NaCl, 0.5% NP-40) supplemented with the protease inhibitor cocktail (Roche, Basel, Switzerland). Cell lysates were centrifuged at 1.2 × 10^4^ rpm for 20 min at 4°C. Immunoprecipitation experiments were performed using Dynabeads Protein G (Thermo Fisher Scientific) according to the manufacturer's instructions. Cell fractionation assays were done using Nuclear and Cytoplasmic Extraction Reagents following the manufacturer's instructions (Pioneer Biotechnology, Xi'an, China).

### Cell proliferation assays, cell cycle analysis and apoptosis assay

For cell counting, 5 × 10^3^ (MCF10A) or 1 × 10^4^ (T47D) cells were seeded in a 24-well plate (Corning, NY, USA) in triplicate. Cell numbers were counted daily for six consecutive days. Proliferation curves were made based on cell numbers from three independent experiments. For cell cycle analysis, 1 × 10^6^ cells were fixed overnight in 70% ethanol and stained using 10 μg/ml Propidium Iodide (PI, Sigma-Aldrich) and 50 μg/ml RNase A (Sigma-Aldrich). Data were analysed by flow cytometry (BD Biosciences, San Jose, CA). For apoptosis assay, 1 × 10^5^ cells were collected and stained with PI and Annexin V by using Apoptosis Detection Kit (KeyGEN Biotech, Nanjing, China). Data were analysed by flow cytometry (BD Biosciences).

### Immunofluorescence

CRB3 antibody (013835) was obtained from Sigma-Aldrich. E-cadherin (ab1416) antibody was purchased from Abcam. YAP (#4912) was obtained from Cell Signaling Technology. ZO-1(339100) antibody and the goat anti-rabbit Alexa Fluor 488-conjugated (A11008) or donkey anti-mouse/rabbit Alexa Fluor 546-conjugated (A10036 and A10040) secondary antibodies were purchased from Thermo Fisher Scientific. Immunofluorescence was performed as previously described.^57^

### Immunohistochemistry and real-time PCR

Immunohistochemistry and real -time PCR were done as previously described.^56^ Primers for real-time PCR are as follows:

CRB3-F, 5′-CTTCTGCAAATGAGAATAGCACTGT-3′

CRB3-R, 5′-GAAGACCACGATGATAGCAGTGA-3′

CTGF-F, 5′-AGGTGTGGCTTTAGGAGCAG-3′

CTGF-R, 5′-TCTTGATGGCTGGAGAATGC-3′

CYR61-F, 5′- TGGAACTGGTATCTCCACACG-3′

CYR61-R, 5′-TACACTGGCTGTCCACAAGG-3′

Kibra-F, 5′-CACAAGGATGGTCTCGTGCTG-3′

Kibra-R, 5′-GCTGTGGCCAATGCCCTTA-3′

FRMD6-F, 5′-GTCAGCCCAGACATGTGCATC-3′

FRMD6-R, 5′-GGAGGTCTTTGGTTTCCGACATA-3′

YAP-F, 5′-CCGTTTCCCAGACTACCTTG-3′

YAP-R, 5′-CAGACTTGGCATCAGCTCCT-3′

GAPDH-F, 5′-CTCCTCCACCTTTGACGCTG-3′

GAPDH-R, 5′-TCCTCTTGTGCTCTTGCTGG-3′.

### Cell adhesion assay

5 × 10^3^ cells were plated in each well of a 24-well cell culture plate (Corning). Cells were left to adhere for 120 min. Non-adherent cells were gently removed and the attached cells were washed with PBS. Then the attached cells were fixed in 95% ethanol for 15 min and stained with 0.4% crystal violet for 30 min. The number of adhesive cells was calculated as the mean of the cell numbers in three randomly selected microscopic fields (Nikon, Japan).

### BrdU incorporation

Cells were cultured to desired confluence, followed by incubation in culture medium with 10 μM BrdU (BD Biosciences) for 3 h. Cells were collected, and fixed with 65% ethanol for 1 h at 4 °C. After cells were washed with PBS, HCl (2N) was used to denature DNA for 20 min at RT. Then cells were neutralized with 0.1 M sodium borate (pH 8.5) for at least 2 min at RT, rinsed with PBS, and incubated with BrdU antibody (BD Biosciences) for 30 min at RT in the dark. Data were analysed by flow cytometry (BD Biosciences).

### 3D culture of MCF10A cells

MCF10A cells were plated at 2 × 10^3^ per well into 3D culture plates within the four-well chamber slide system (177437, Corning) and cultured in growth factor-reduced Matrigel matrix (BD Biosciences). 3D culture of MCF10A cells and staining of cleaved caspase 3 (1:200, #9664, Cell Signaling Technology) were performed as previously described.^29^ Images were taken and analysed on the sixteenth day of cell culture. Cells were stained on the eighth day of cell culture.

### Mammary fat pad xenograft experiments

All animal procedures were complied with the guidelines of the Institutional Animal Use and Care Committee of Xi'an Jiaotong University. Four-week-old SCID female mice were bred at the specific pathogen-free animal facility. For xenograft studies, 2 × 10^6^ MDA-MB-231 cells (diluted in 100 μl PBS) were injected into the mammary fat pad of the mice on both flanks. Twenty-five days after cell injection, tumours were dissected, weighed, photographed and fixed in 4% paraformaldehyde for hematoxylin-eosin staining and immunohistochemistry. Immunohistochemistry was done as previously described.^56^

### Statistical analysis

Statistical analyses were performed using the SPSS 13.0 (SPSS Inc., USA). Values were expressed as mean±S.D. Comparisons between two groups were conducted using the unpaired Student's *t*-test. One-way ANOVA was used for multiple comparisons. All statistical tests were two-sided. *P*<0.05 was considered as indicating statistical significance. All data shown are from experiments that were performed at least three times with similar results on each occasion.
