# Supplementary material for: CRB3 regulates contact inhibition by activating the Hippo pathway in mammary epithelial cells
Source: Cell Death Dis. 2017 Jan 12;8(1):e2546–. doi: 10.1038/cddis.2016.478 (PMC5386381; doi:10.1038/cddis.2016.478)
Supplement: Supplementary Material [file cddis2016478x1.docx]

**Author contributions**

XN Mao and PP Li performed most of experiments, analyzed the data and assembled the Figures, such as Figure 1, Figure 5, Figure 6, Figure S3, Figure S4 and Figure S5. YC Wang and ZY Liang prepared Figure S1, Figure S2, Figure S6 and designed the model in Figure 7 and Figure S7. J Liu and J Li prepared Figure 2 and conducted some immunoblot analysis in Figure 3 and Figure 4. YN Jiang, G Bao, L Li, and BF Zhu conducted remaining experiments in Figure 3 and Figure 4 and assembled the Figure 3 and Figure 4. Y Ren and XH Zhao collected the breast cancer tissues and their adjacent normal breast tissues which were used in Figure S1. Y Ren, XH Zhao, JM Zang, and Y Liu gave many scientific advices of the experiments design and statistical analysis. JM Zhang also provided some plasmids in the experiments, such as plasmids of Kibra knockdown or overexpression. XN Mao and PJ Liu wrote the manuscript. J Yang and PJ Liu designed experiments, supervised all experiments and helped write the manuscript.

**Supplementary Figure Legends**

**Figure S1** CRB3 expression is significantly down-regulated in breast cancer tissues and breast cancer cells. (**a**) CRB3 expression was detected using IHC, and positive or negative staining is shown in images. (**b**) CRB3 expression was examined in breast cancer tissues and adjacent normal breast tissues. (**c**) The image showed the percentage of CRB3 expression in breast cancer tissues and adjacent normal breast tissues. ****P*< 0.001. Data are mean ± SD. (**d and e**) The CRB3 mRNA and protein expression levels in immortalized mammary epithelial cells (MCF10A and MCF12A) and breast cancer cells (MCF7, T47D, MDA-MB-231 and MDA-MB-453) were examined by real-time PCR and immunoblotting, respectively. GAPDH was used as loading control.

**Figure S2** Overexpression of CRB3 inhibits cell proliferation in MDA-MB-453 cells. (**a and b**) The overexpression efficiencies of CRB3 were determined by immunoblot analysis and real time-PCR. ****P* < 0.001. Data are mean ± SD. (**c** ) The proliferation of MDA-MB-453 cells was evaluated for 6 successive days using a cell proliferation assay. **P* < 0.05, ***P* < 0.01. Data are mean ± SD. (**d**) A cell cycle analysis was used to measure the cell cycle distribution of cells grown under sparse and confluent conditions. **P* < 0.05. Data are mean ± SD. (**e**) BrdU incorporation was examined to reveal DNA synthesis in cells grown under sparse and confluent conditions. **P* < 0.05. Data are mean ± SD. (**f**) The protein expression levels in the indicated cells were detected by immunoblotting.

**Figure S3** CRB3 regulates cell proliferation. Cell proliferation was measured in MCF10A and T47D cells grown under sparse and confluent conditions using a cell cycle analysis. (**a**) The quantitative analysis of the cell cycle distribution of MCF10A cells is shown. (**b and c**) The quantitative analysis of the cell cycle distribution of T47D cells is shown. **P* < 0.05, ***P* < 0.01. Data are mean ± SD.

**Figure S4** CRB3 overexpression promotes cell apoptosis in MDA-MB-453 cells. (**a and b**) Cell apoptosis assay was used to detect the cell apoptosis. ***P* < 0.01. Data are mean ± SD. (**c**) An immunoblot analysis was used to detect the expression of apoptosis-related proteins. Cas 3, caspase 3; cas 9, caspase 9.

**Figure S5** Mst2 and Lats1 were silenced with siRNAs in shCRB3 cells. MCF10A cells were infected with a lentivirus (shCRB3) or vector control lentivirus to knock down CRB3. (**a**) shCRB3 cells were transfected with the siRNAs to inhibit Mst2 expression. The expression levels of indicated proteins were detected by immunoblotting. (**b**) shCRB3 cells were transfected with the siRNAs to knock down Lats1 expression. The expression levels of indicated proteins were detected by immunoblotting.

**Figure S6** YAP expression was silenced with siRNAs in MCF10A cells. MCF10A cells were infected with a lentivirus (shCRB3) or vector control lentivirus to knock down CRB3 expression. shCRB3 cells were transfected with the indicated siRNA or negative control (NC) to silence YAP expression. The efficiency of silencing YAP mRNA expression was analysed using real-time PCR. ***P* < 0.01, ****P* < 0.001. Data are mean ± SD.

**Figure S7** The schematic model shows the relationship between CRB3 expression and the Hippo pathway in mammary epithelial cells and breast cancer cells grown under sparse and confluent conditions. (**a1**) CRB3 is expressed at high levels and the Hippo pathway is inactivated (Hippo off) in mammary epithelial cells grown under sparse conditions. CRB3 is expressed at low levels and the Hippo pathway is activated (Hippo on) in the confluent mammary epithelial cells. The reason for the low CRB3 expression in confluent mammary epithelial cells may be that CRB3 is regulated by endocytosis or Retromer. (**a2**) CRB3 is expressed at low levels and the Hippo pathway is inactivated (Hippo off) in breast cancer cells, regardless of the degree of confluence.

**Table S1** Relationship between CRB3 expression and clinicopathologic parameters of breast cancer patients

| Variables | No. | Expression Levels | | χ^2^ | *P* |
| --- | --- | --- | --- | --- | --- |
|  |  | Negative | Positive |  |  |
| Age(years) |  |  |  | 1.834 | 0.176 |
| ≥50 | 24 | 21 | 3 |  |  |
| <50 | 17 | 11 | 6 |  |  |
| Histologic grade |  |  |  | 0.484 | 0.487 |
| Ⅱ | 16 | 11 | 5 |  |  |
| Ⅲ | 24 | 20 | 4 |  |  |
| missing | 1 |  |  |  |  |
| Clinical stage |  |  |  | 1.567 | 0.211 |
| 0~2 | 27 | 19 | 8 |  |  |
| 3 | 14 | 13 | 1 |  |  |
| Tumor size(cm) |  |  |  | 3.876 | 0.049 |
| <2 | 7 | 3 | 4 |  |  |
| ≥2 | 34 | 29 | 5 |  |  |
| Tumor site |  |  |  |  | 1.000 |
| Left | 21 | 16 | 5 |  |  |
| Right | 20 | 16 | 4 |  |  |
| Lymph node involvement |  |  |  |  | 0.130 |
| Negative | 20 | 18 | 2 |  |  |
| Positive | 21 | 14 | 7 |  |  |
