# Supplementary figures and images for: CRB3 regulates contact inhibition by activating the Hippo pathway in mammary epithelial cells
Source: Cell Death Dis. 2017 Jan 12;8(1):e2546–. doi: 10.1038/cddis.2016.478 (PMC5386381; doi:10.1038/cddis.2016.478)

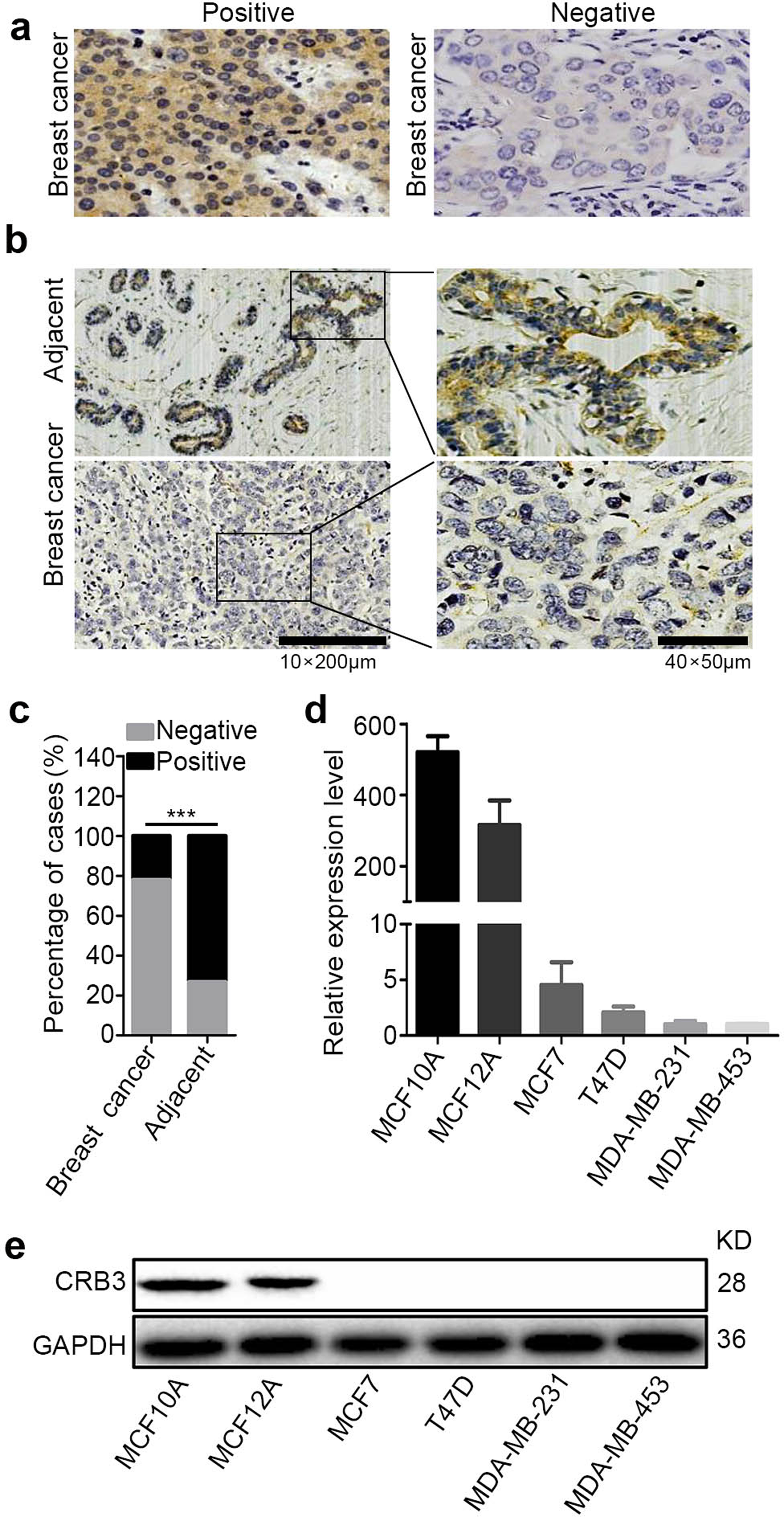

Supplement: Supplementary Figure S1 [file cddis2016478x2.tif]

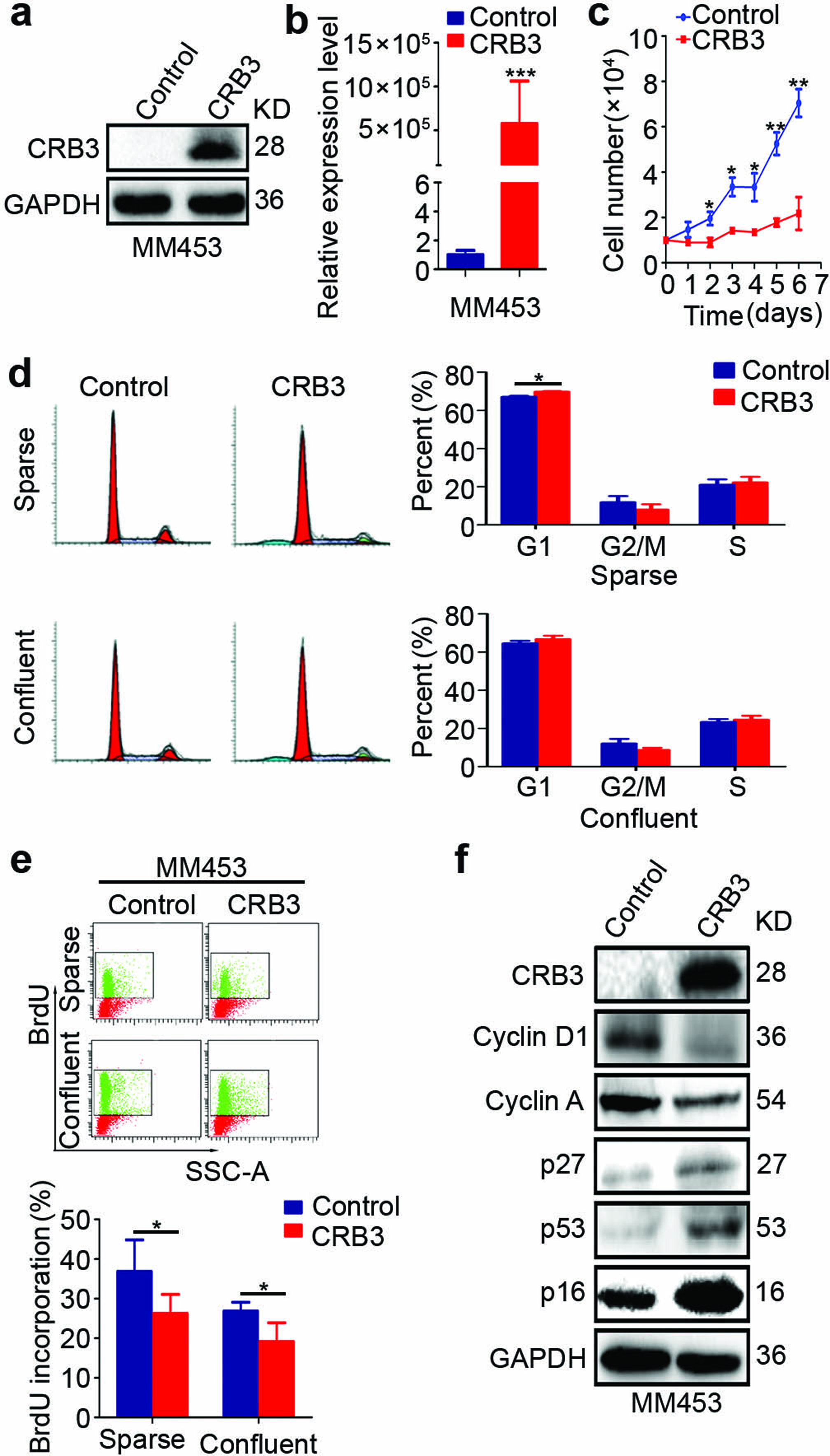

Supplement: Supplementary Figure S2 [file cddis2016478x3.tif]

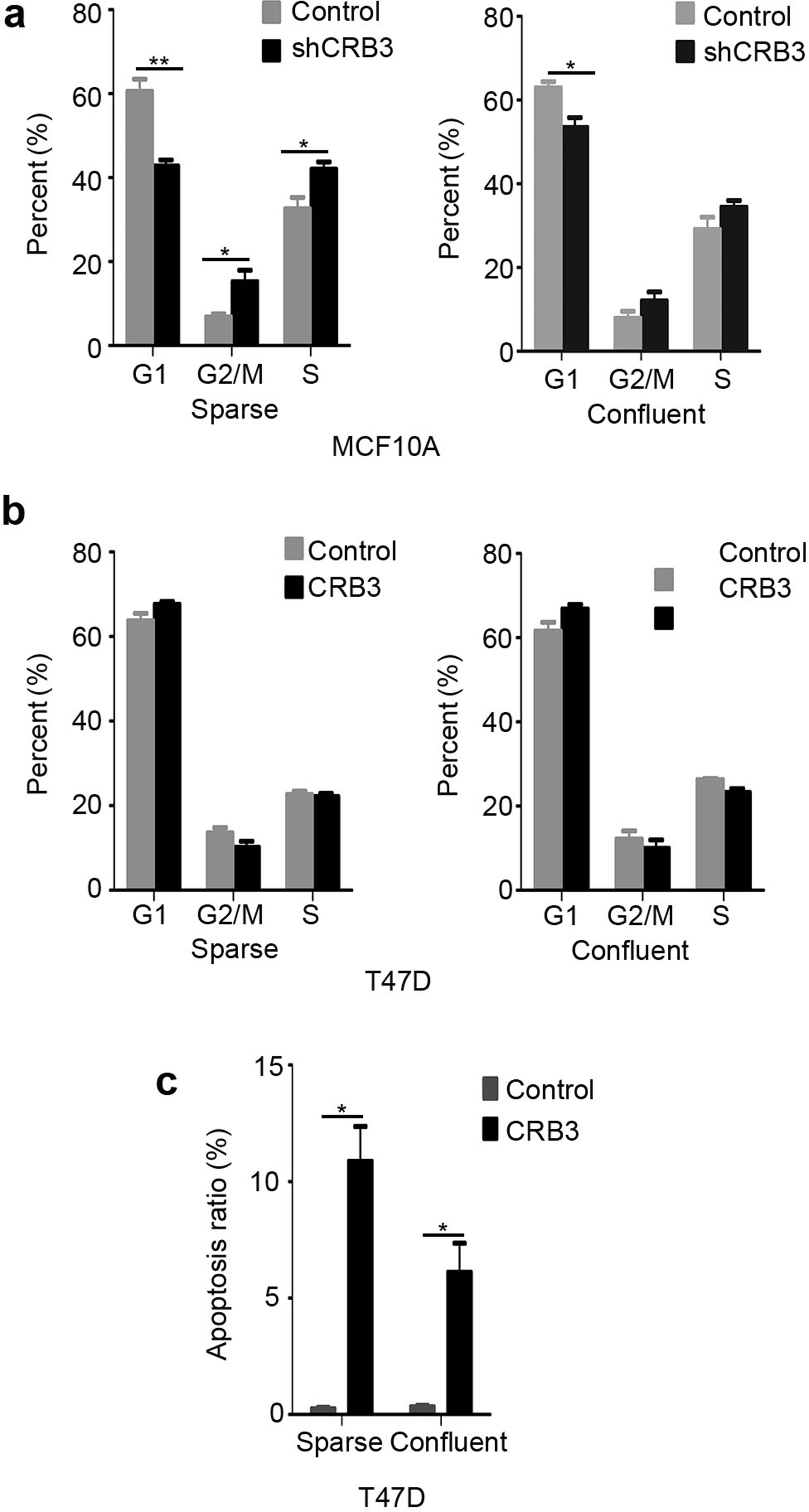

Supplement: Supplementary Figure S3 [file cddis2016478x4.tif]

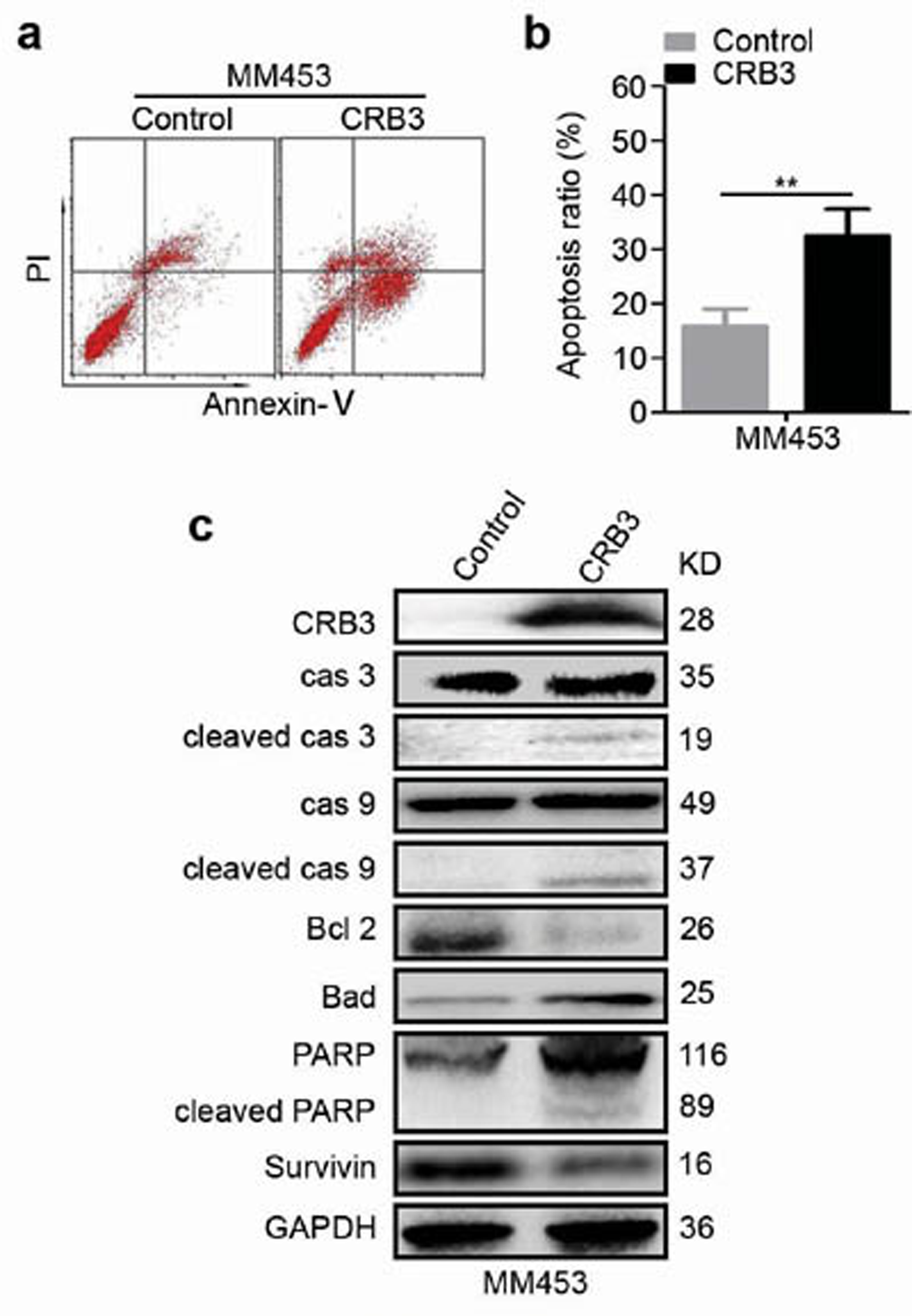

Supplement: Supplementary Figure S4 [file cddis2016478x5.tif]

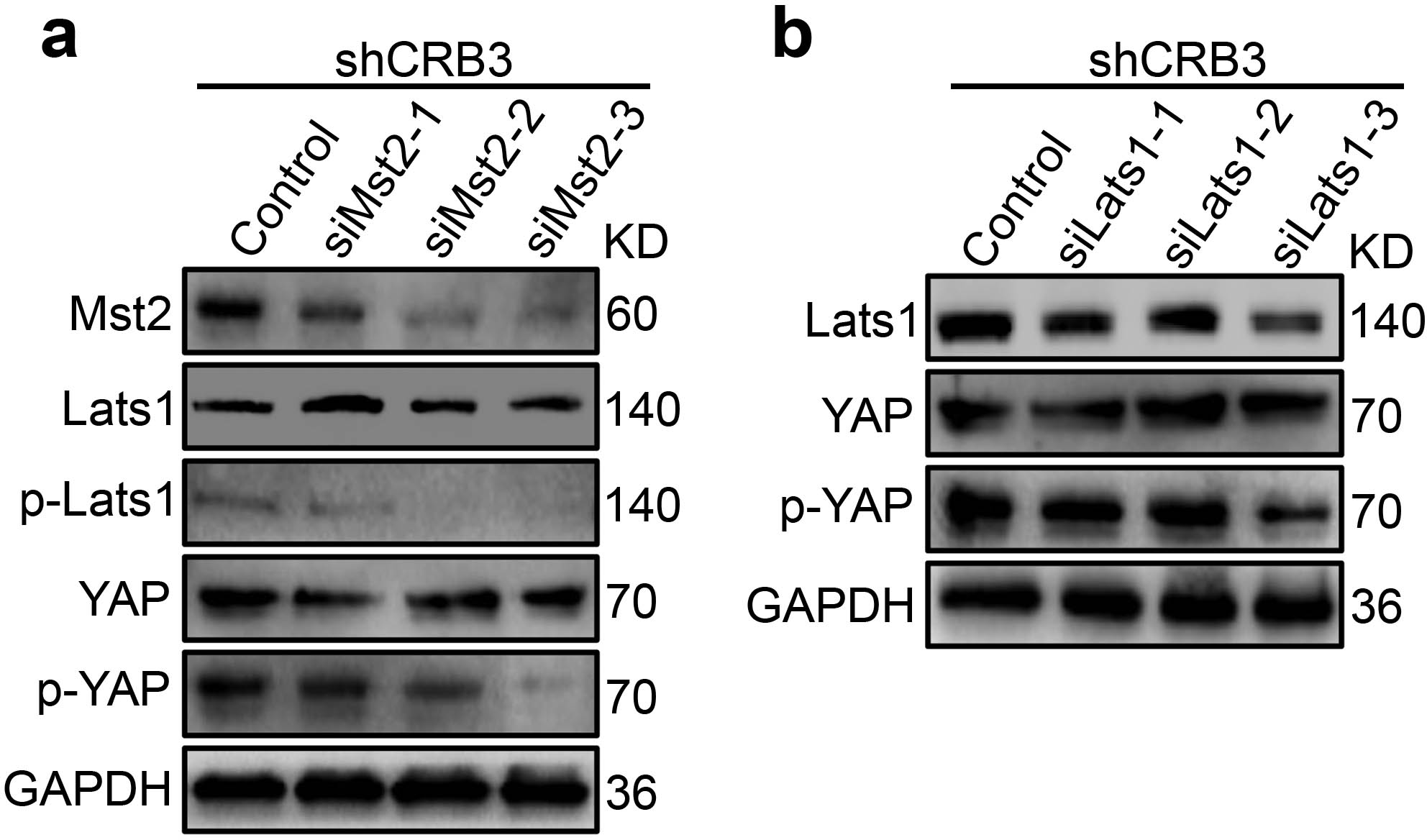

Supplement: Supplementary Figure S5 [file cddis2016478x6.tif]

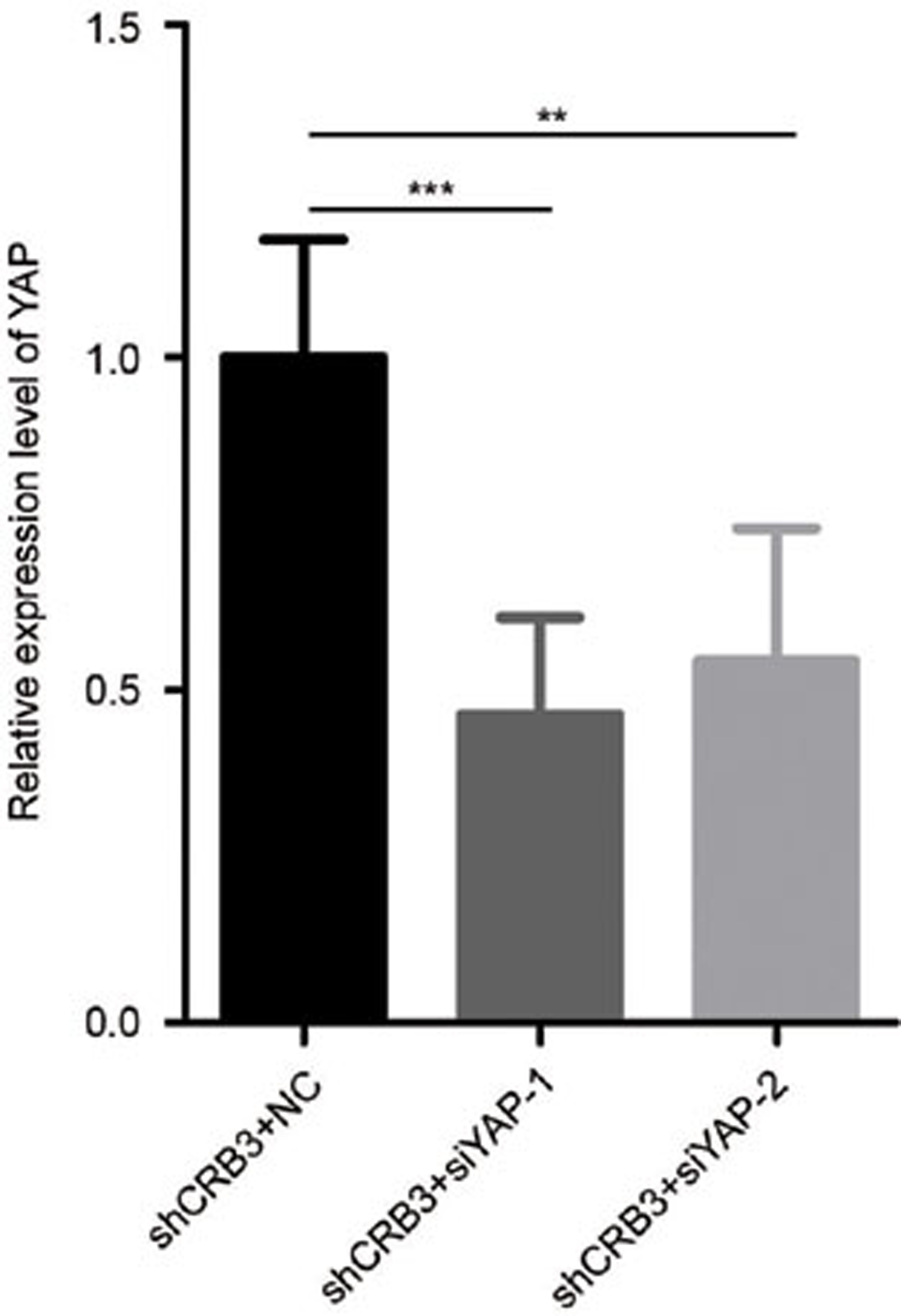

Supplement: Supplementary Figure S6 [file cddis2016478x7.tif]

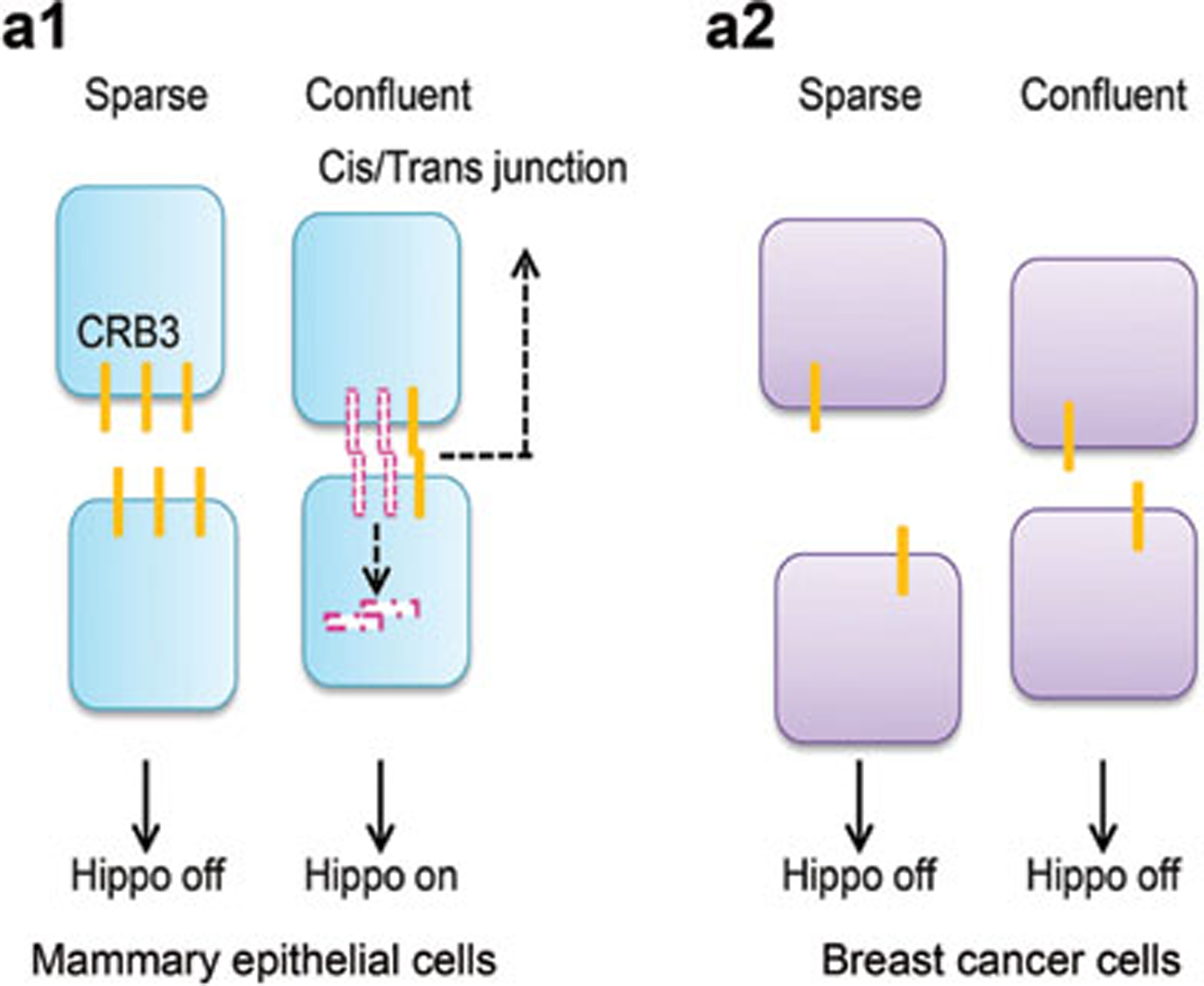

Supplement: Supplementary Figure S7 [file cddis2016478x8.tif]
